# Machine learning technology in the classification of glaucoma severity using fundus photographs

**DOI:** 10.1038/s41598-025-11697-1

**Published:** 2025-07-18

**Authors:** Sukhumal Thanapaisal, Passawut Uttakit, Worapon Ittharat, Pukkapol Suvannachart, Pawasoot Supasai, Pattarawit Polpinit, Prapassara Sirikarn, Panawit Hanpinitsak

**Affiliations:** 1 Department of Ophthalmology, Faculty of Medicine, Khon Kaen University, Khon Kaen, 40002 Thailand; 2 KKU Glaucoma Center of Excellence, Department of Ophthalmology, Faculty of Medicine, Khon Kaen University, Khon Kaen, Thailand; 3 Department of Computer Engineering, Faculty of Engineering, Khon Kaen University, Khon Kaen, Thailand; 4 Department of Ophthalmology, Faculty of Medicine, Mahasarakham University, Mahasarakham, Thailand; 5 Department of Epidemiology and Biostatistics, Faculty of Public Health, Khon Kaen University, Khon Kaen, Thailand

**Keywords:** Machine learning, Glaucoma, Classification, Screening, Glaucoma, Medical imaging, Diagnosis

## Abstract

This study evaluates the performance of a machine learning model in classifying glaucoma severity using color fundus photographs. Glaucoma severity grading was based on the Hodapp-Parrish-Anderson (HPA) criteria incorporating the mean deviation value, defective points in the pattern deviation probability map, and defect proximity to the fixation point. The dataset of 2,940 fundus photographs from 1,789 patients was matched with visual field tests and equally classified into three classes: normal, mild-moderate, and severe glaucoma stages. The EfficientNetB7, a convolutional neural network model, was trained on these images using transfer learning and fine-tuning techniques. The model achieved an overall accuracy of 0.871 (95% CI, 0.822–0.919). For normal, mild-moderate, and severe classes, the area under the curve (AUC) values were 0.988, 0.932, and 0.963; sensitivity 0.903, 0.823, and 0.887; and specificity 0.960, 0.911, and 0.936, respectively. Analysis of the confusion matrix revealed the impact of structural-functional relationships in glaucoma on model performance. In conclusion, the EfficientNetB7 demonstrated high accuracy in classifying glaucoma severity based on the HPA criteria using fundus photographs, offering potential for clinical application in glaucoma diagnosis and management.

**Supplementary Information:**

The online version contains supplementary material available at 10.1038/s41598-025-11697-1.

## Introduction

Glaucoma is the leading cause of irreversible blindness worldwide. The prevalence is estimated to increase to 111.8 million in 2040^1^. Glaucoma screening plays an important role in early detection and the prevention of visual loss. Characterized by damage to the optic nerve head (ONH) shown in fundus photographs, retinal nerve fiber layer (RNFL) defects in optical coherence tomography (OCT) and visual field defects in standard automated perimetry (SAP), glaucoma detection could be performed by combining these tools for more accurate diagnosis. On the contrary, only fundus photographs are available for most areas of glaucoma screening, especially in rural locations.

Machine learning (ML) technology has been applied in the detection of glaucoma using color fundus photographs^2–4^, OCT imaging^5–7^, and OCT angiography^8^, demonstrating high accuracy, as confirmed by meta-analysis^9^. Moreover, the combination of visual field tests and OCT images in developing the multimodal model revealed a superior performance to the single test^10^.

For glaucoma severity classification, ML showed a high, stable performance in the use of a combined convolutional neural network (CNN) model via fundus photographs^7,11^ and OCT ONH scans^12^. The performance decreased when incorporating the demographic data of patients into the model^13^. However, most of these studies classified the severity of glaucoma by the mean deviation (MD) value of the visual field only.

Hodapp-Parrish-Anderson classification (HPA) is based on two criteria. The first criterion is the overall extent of damage calculated by using both the MD value and the number of defective points in the pattern deviation probability map, whereas the second criterion is based on the proximity of the defect to the fixation point. This system divides early, moderate, and severe glaucomatous visual field defects^14^ and recognizes subtle nerve damage to diagnose early glaucoma^15^. The purpose of this study is to evaluate the performance of ML technology in classifying glaucoma severity using color fundus photographs and grading the severity by HPA classification.

## Methods

This retrospective study was approved by the Human Research Ethics Committee (HREC) of the hospitals and conducted in accordance with the tenets of the Declaration of Helsinki. The Khon Kaen University Ethics Committee for Human Research and the Mahasarakham University Ethics Committee for Research involving Human Subjects waived the requirement for informed consent. Data were collected from patients attending the ophthalmology clinics at Srinagarind Hospital and Suddhavej Hospital between January 2016 and October 2023. All images were anonymized to ensure confidentiality.

### Participants

Glaucoma patients and normal participants aged 18 years or older were included in the study. Diagnosis of glaucoma patients was based on the detection of vertically enlarged cupping, RNFL defects in fundus photographs or OCT, and Anderson’s criteria of glaucomatous visual field defects. The exclusion criteria were glaucoma suspects, patients with myopia, or hyperopia more severe than − 3.0 D or + 3.0 D, non-glaucomatous optic neuropathies, e.g., inflammatory, compressive, ischemic, congenital, vitreoretinal diseases, e.g., age-related macular degeneration, diabetic retinopathy, retinal vascular occlusion, epiretinal membrane, and poor-quality fundus images or visual field tests.

### Dataset collection and labeling

The inclusion criteria at the input data level consisted of color fundus images of the included participants acquired by two digital fundus cameras (Nonmyd Alpha WX 3D and Nonmyd AF, Kowa Optimed, Tokyo, Japan). The images were captured in two modes of the internal fixation target: central and disc positions. The fundus photographs were obtained from participants with unilateral and bilateral glaucoma. In some cases, a pair of fundus photographs from both eyes was used, while others, only a single fundus photograph was included. All the visual fields (VF) were performed on SAP using the Swedish interactive thresholding algorithm (SITA-Standard) of central 24 − 2 or 30 − 2 perimetry (Humphrey field analyzer II, Carl Zeiss Meditec, Dublin, CA, USA). Visual field testing was considered unreliable when the fixation losses were higher than 20%, the false-positive rate higher than 15%, or the false-negative rate higher than 33%. Reliable visual field data within six months of the fundus photograph were selected.

The pairing of fundus photographs and VF test results was reviewed by three glaucoma specialists (S.T., W.I., and Pu.S.). These specialists performed ground tooth labeling by grading the severity of each fundus photograph. The photographs were classified into four groups of severity: normal, mild, moderate, and severe, according to the HPA criteria^16^, as described in Data S1 (see Supplementary Data S1 online). Notably, optic disc morphology and OCT parameters were not used to determine the glaucoma severity. No glaucomatous structural change or VF defect was present in the normal group. The reviewers classified the defect using the MD value, point locations, and point severity from the criteria. Any questionable results were resolved through discussion with three reviewers. When two of the three glaucoma specialists could not reach an agreement, a senior glaucoma expert (Pa.S.) was asked to reach a consensus. Fundus photographs without consensus were excluded from the dataset.

### Image preprocessing and model development

**Fig. 1:**
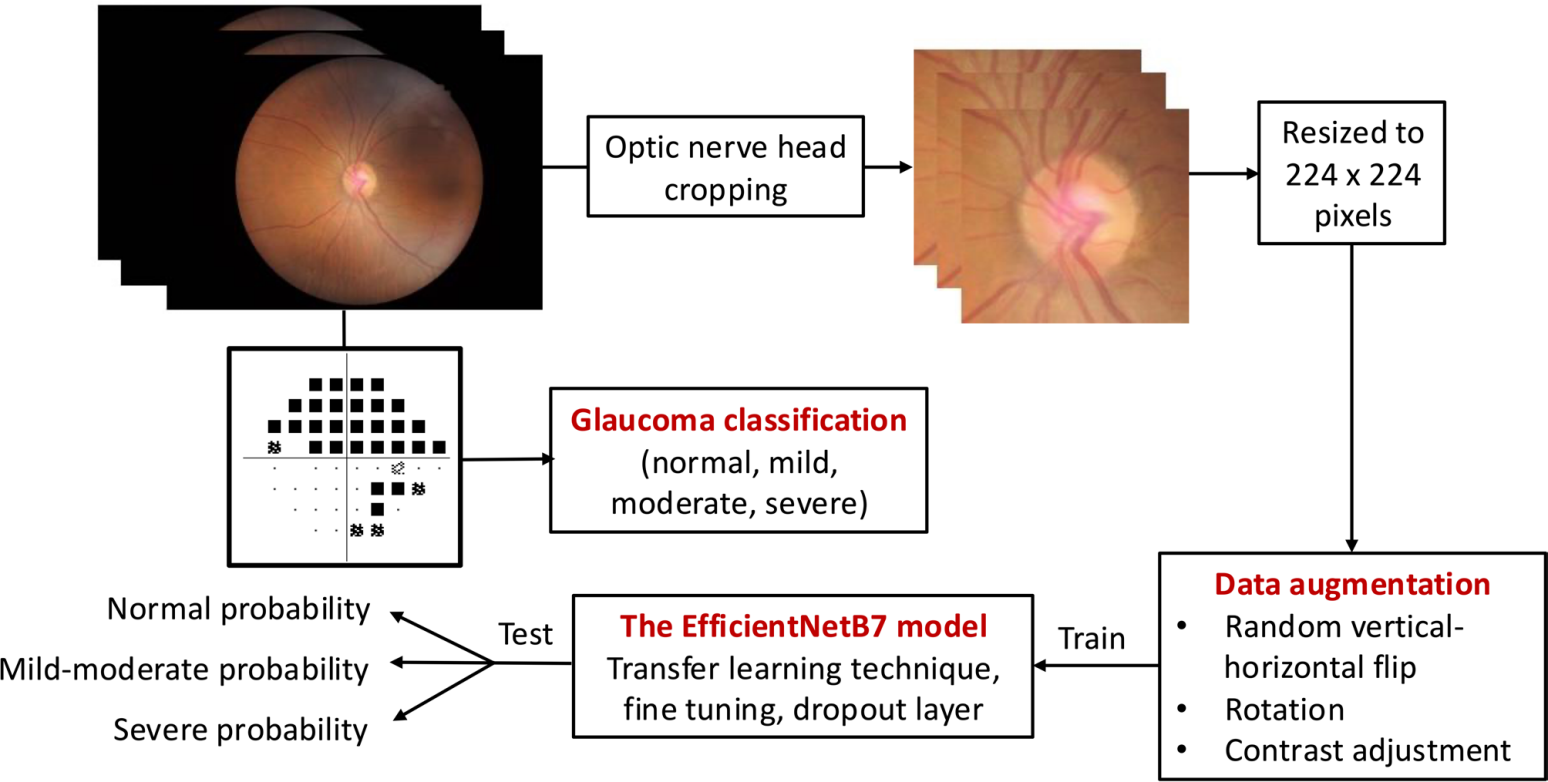
Image processing flow and workflow of the study.

All fundus images were preprocessed before input into the CNN model. First, the fundus images were cropped at the ONH region because this area represents glaucomatous damage. All images were resized to 224 × 224 pixels. Data augmentation was performed by random vertical and horizontal flip, rotation, and contrast adjustment. The EfficientNetB7 model^17^, pre-trained on the ImageNet^18^, was used as the base CNN architecture. Dropout layer was applied to prevent overfitting, and the last fully connected layer (classification layer) was then added to predict the glaucoma severity levels. Transfer learning was applied by freezing all convolutional layers of EfficientNetB7 and training only the newly added classification layer on the fundus image dataset. Subsequently, fine-tuning was performed on the same dataset by unfreezing and training only the final 39% of the layers (layers beyond index 500), while the remaining 61% of the layers remained frozen. This allows the model to adapt high-level features to the fundus image domain while preserving the general representations learned from ImageNet. The EfficientNetB7 model outputs the prediction of normal, mild-moderate, or severe glaucoma. Figure 1 demonstrates the image processing flow and workflow of the study.

### Statistical analysis

The accuracy and area under the curve (AUC) reported by the confusion matrix and ROC curve were used as outcome measures to evaluate the diagnostic performance of the CNN model in classifying the stage of glaucoma. The sensitivity, specificity, precision (positive predictive value), negative predictive value, and F1 score ((precision + recall)/2) overall and at each stage of glaucoma were reported. Statistical analysis was performed using Stata statistical software (v10.1, StataCorp, Texas, USA) and Python (v.3.10.12, Python Software Foundation).

Ten-fold cross-validation with three-way split (train/validation/test) was performed to evaluate the effectiveness of the CNN model. Specifically, the training and validation sets from Table 1 (2,568 + 186 = 2,754 images) were combined and then divided into 10 folds. In each iteration, approximately 90% of these images (2,477 out of 2,754) were used for training, while the remaining 10% were used for validation. The held-out test set (186 images) was not involved in cross-validation and was reserved exclusively for final model evaluation to prevent overfitting.

**Table 1:** Distribution of the dataset and patient characteristics.

	Normal	Mild-moderate	Severe
Training set			
Images, n	856	799	913
Eyes, n	856	799	913
Right/left	448/408	391/408	447/466
Patients, n	500	451	466
Age, mean (SD), yrs	60.9 (14.3)	68.1 (12.1)	67 (15.2)
Sex; male, n (%)	243 (48.6)	235 (50.1)	252 (55.4)
Validation set			
Images, n	62	62	62
Eyes, n	62	62	62
Right/left	31/31	30/32	39/23
Patients, n	62	62	62
Age, mean (SD), yrs	60.2 (14.1)	65.5 (12.1)	63.8 (15.9)
Sex; male, n (%)	30 (48.3)	28 (45.2)	38 (61.3)
Testing set			
Images, n	62	62	62
Eyes, n	62	62	62
Right/left	29/33	28/34	34/28
Patients, n	62	62	62
Age, mean (SD), yrs	60.4 (12.0)	68.4 (11.2)	67.9 (15.2)
Sex; male, n (%)	31 (50)	27 (43.5)	39 (62.9)

## Results

### Distribution of the dataset

A total of 2,940 fundus photographs from 1,789 patients were included. The distribution of the dataset and patient characteristics are demonstrated in Table 1. The fundus photographs were classified into four groups, but the number of images was insufficient to optimize the model. Finally, the dataset was reclassified into three groups: normal, mild-moderate, and severe. Images were divided into training, validation, and testing sets.

### Performance of the model in the validation set

**Fig. 2:**
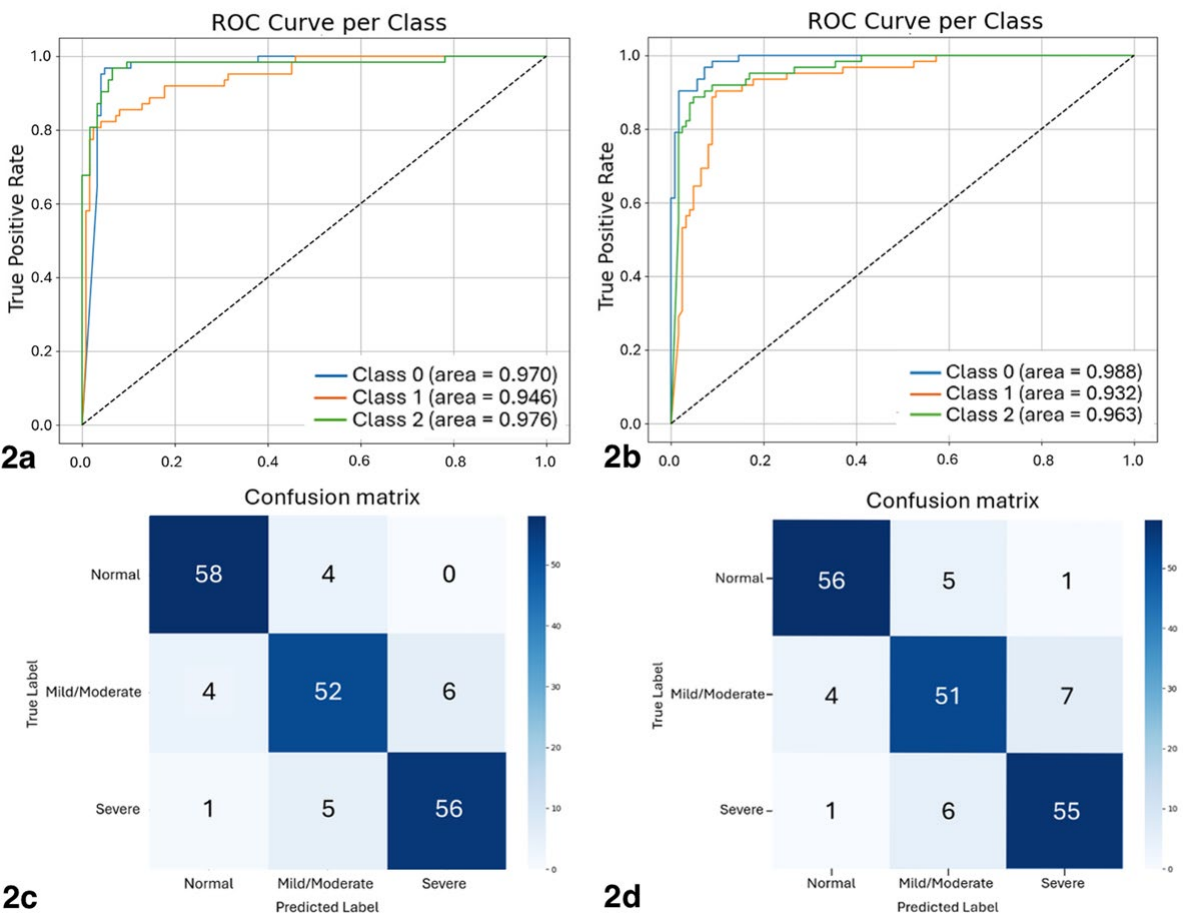
Area under the receiver operator characteristic curves (AUCs) of the normal, mild-moderate, and severe classes in the validation (2a) and testing (2b) sets. Confusion matrix demonstrating the diagnostic performance (raw count) of the validation (2c) and testing (2d) sets.

The diagnostic performance of the model, EfficientNetB7, is shown in Table 2. In the validation set, the model provided an overall accuracy of 0.892 (95% CI, 0.844–0.935). Based on the normal, mild-moderate, and severe classes, the accuracy in each class was 0.936, 0.839, and 0.903, respectively. The AUC values were 0.970, 0.946, and 0.976, respectively (Fig. 2a). Sensitivity in each class was 0.936, 0.839, and 0.903, with the specificity being 0.960, 0.927, and 0.952, respectively. The confusion matrix for Epoch 72 demonstrated the results of three image classifications in the validation set (Fig. 2c).

**Table 2:** Performance of the machine learning model in glaucoma classification.

Parameters	Glaucoma classification			Overall
	Normal	Mild-moderate	Severe	
Validation set				
Accuracy	0.936 (0.867–0.985)	0.839 (0.745–0.924)	0.903 (0.821–0.967)	0.892 (0.844–0.935)
AUC	0.970	0.946	0.976	0.965
Sensitivity	0.936 (0.868–0.985)	0.839 (0.734–0.926)	0.903 (0.822–0.981)	
Specificity	0.960 (0.863–0.984)	0.927 (0.881–0.966)	0.952 (0.830–0.967)	
Precision	0.921 (0.850–0.982)	0.853 (0.754–0.936)	0.903 (0.826–0.969)	
NPV	0.968 (0.919–0.991)	0.920 (0.858–0.961)	0.952 (0.898–0.982)	
F1 score	0.928 (0.873–0.969)	0.846 (0.769–0.912)	0.903 (0.842–0.952)	0.892 (0.846–0.934)
Testing set				
Accuracy	0.903 (0.824–0.969)	0.823 (0.726–0.909)	0.887 (0.800–0.955)	0.871 (0.822–0.919)
AUC	0.988	0.932	0.963	0.962
Sensitivity	0.903 (0.828–0.968)	0.823 (0.719–0.918)	0.887 (0.806–0.962)	
Specificity	0.960 (0.908–0.987)	0.911 (0.847–0.955)	0.936 (0.877–0.972)	
Precision	0.918 (0.843–0.983)	0.823 (0.732–0.912)	0.873 (0.784–0.948)	
NPV	0.952 (0.898–0.982)	0.911 (0.847–0.955)	0.943 (0.886–0.977)	
F1 score	0.911 (0.850–0.958)	0.823 (0.737–0.887)	0.880 (0.815–0.935)	0.871 (0.818–0.914)

AUC, area under the curve; NPV, negative predictive value.

### Performance of the model in the testing set

In the testing set, the model provided an overall accuracy of 0.871 (95% CI, 0.822–0.919). Based on the normal, mild-moderate, and severe classes, the accuracy in each class was 0.903, 0.823, and 0.887, respectively. The AUC was 0.988, 0.932, and 0.963, respectively (Fig. 2b). Sensitivity in each class was 0.903, 0.823, and 0.887, with the specificity being 0.960, 0.911, and 0.936, respectively. The confusion matrix showed the results of the image classification in the testing set (Fig. 2d). The average inference time per model image was 741 milliseconds, based on running the Google Colab environment with a CPU configuration of two cores, Intel Xeon (2.20 GHz), and 13GB of available RAM. We were not able to report the 95% CI of the AUC calculated from the normal model training technique because the training, evaluation, and testing were performed only once. Nevertheless, the 95% CI was calculated by 10-fold cross-validation.

### Performance of the model by 10-fold cross-validation

Using a 10-fold cross-validation technique, the overall accuracy of the model was 0.882 (0.870–0.895) in the validation set and 0.866 (0.856–0.874) in the testing set. The model achieved AUC of 0.980 (0.974–0.985), 0.943 (0.932–0.954), and 0.978 (0.972–0.984) for normal, mild-moderate, and severe classes in the validation set. For the internal test set, the model demonstrated AUC of 0.987 (0.984–0.989), 0.924 (0.913–0.933), and 0.961 (0.954–0.966), which are comparable to those obtained using the normal model training technique. The model performance using 10-fold cross-validation technique was demonstrated in Table 3.

**Table 3:** Performance of the machine learning model in glaucoma classification by 10-fold cross-validation.

Parameters	Glaucoma Classification			Overall
	Normal	Mild-moderate	Severe	
Validation set				
Accuracy	0.926 (0.901–0.951)	0.830 (0.791–0.868)	0.888 (0.861–0.915)	0.882 (0.870–0.895)
AUC	0.980 (0.974–0.985)	0.943 (0.932–0.954)	0.978 (0.972–0.984)	0.967 (0.961–0.972)
Sensitivity	0.926 (0.901–0.951)	0.830 (0.792–0.868)	0.889 (0.861–0.915)	
Specificity	0.946 (0.932–0.960)	0.916 (0.899–0.932)	0.963 (0.956–0.969)	
Precision	0.897 (0.873–0.921)	0.821 (0.793–0.848)	0.930 (0.919–0.940)	
NPV	0.963 (0.951–0.975)	0.923 (0.906–0.938)	0.941 (0.927–0.954)	
F1 score	0.911 (0.895–0.926)	0.824 (0.802–0.846)	0.909 (0.894–0.922)	0.881 (0.868–0.894)
Testing set				
Accuracy	0.932 (0.913–0.950)	0.832 (0.807–0.856)	0.832 (0.802–0.861)	0.866 (0.856–0.874)
AUC	0.987 (0.984–0.989)	0.924 (0.913–0.933)	0.961 (0.954–0.966)	0.957 (0.951–0.962)
Sensitivity	0.932 (0.917–0.946)	0.832 (0.813–0.851)	0.832 (0.809–0.854)	
Specificity	0.959 (0.955–0.962)	0.895 (0.881–0.909)	0.944 (0.935–0.952)	
Precision	0.919 (0.912–0.925)	0.801 (0.780–0.820)	0.883 (0.866–0.898)	
NPV	0.966 (0.959–0.973)	0.915 (0.906–0.923)	0.919 (0.908–0.928)	
F1 score	0.925 (0.918–0.932)	0.815 (0.803–0.827)	0.856 (0.841–0.871)	0.866 (0.856–0.874)

AUC, area under the curve; NPV, negative predictive value.

### Characteristics of misclassified fundus photographs

The overall misclassified images accounted for 10.75% and 14.5% of validation and testing sets, respectively. Most of the misclassified fundus photographs comprised images predicted to be in the mild-moderate class, whereas the images were actually severe according to the HPA criteria. Another example of misclassified photos was the actual mild-moderate images predicted to be in the severe and normal groups. Fig. S2 demonstrates the characteristics, number, and explanations of all misclassified fundus photographs in the testing sets (see Supplementary Fig. S2 online).

### Visualization of model prediction

**Fig. 3:**
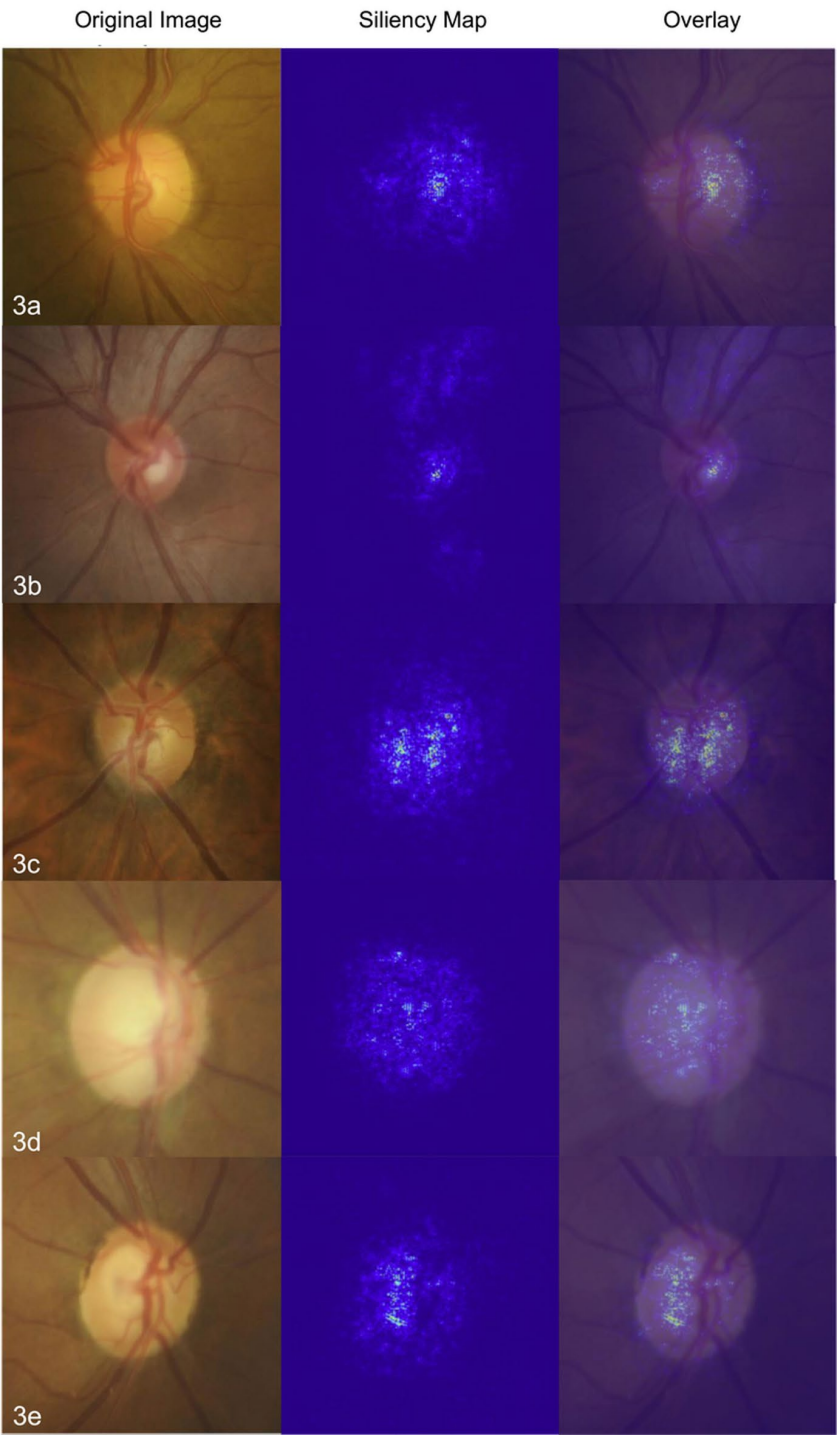
Saliency and overlay visualizations from the testing sets, including normal (3a, 3b), mild-moderate (3c), and severe (3d) glaucoma classes. The model consistently highlights the optic disc cupping region in most images. Notable highlighting of the retinal nerve fiber layer is observed in several cases, particularly in the normal (3b) and mild classes. A misclassified image of an actual mild-moderate case was predicted in severe class (3e).

The results of glaucoma classification were visualized using Gradient-weighted Class Activation Mapping (Grad-CAM). Figure 3 presents the corresponding saliency maps and overlays for each class. The saliency visualizations reveal that, across normal, mild-moderate, and severe glaucoma classes, the model consistently highlights the optic disc cupping region. The intensity of the highlighted area increases when the cupping is deeper and more distinct from the surrounding rim (Fig. 3a and c). In cases of large or advanced cupping, where the distinction between the cup and rim is less apparent, the highlighted areas become more generalized (Fig. 3d). Additionally, the peripapillary retinal nerve fiber layer is highlighted in several cases where it is particularly prominent, as seen in Fig. 3b. An example of misclassification based on the saliency map is shown in Fig. 3e.

## Discussion

In this study, we developed and validated a CNN model, EfficientNetB7, using real-world fundus photographs and visual field tests to classify glaucoma severity according to the HPA classification criteria. The algorithms were trained, validated, and tested by glaucoma and normal datasets, enabling us to differentiate the data into three classes: normal, mild-moderate, and severe glaucoma. The EfficientNetB7 model achieved good performance and showed high sensitivity in all three data classes, proving useful for glaucoma screening by fundus photographs. The results demonstrated that the traditional method of glaucoma severity classification could be used for glaucoma screening, especially in the detection of severe cases needing urgent treatment.

We achieved slightly higher accuracy compared to the previous study^19^, that employed deep learning on color fundus photographs to classify glaucoma into normal, mild, moderate, and severe levels. However, differences in glaucoma classification criteria and number of classes used in that study limit the direct comparability of results. Another study performed a subgroup analysis using ONH scan images and reported high model performance across different glaucoma severity levels^12^. That study reported recall values for the mild, moderate, and severe classes, which differ from the metrics used in our analysis. To the best of our knowledge, this is the only study that classifies glaucoma severity by fundus photographs using ground-truth labeling with the HPA criteria.

As a screening tool, sensitivity was used to evaluate the performance of the tool. We proposed high sensitivity in all classes, with the highest value of 0.903 observed in the normal class. This finding was similar with previous studies, which reported comparable sensitivity values for mild, moderate, and severe classes^12^.

There were misclassified fundus photographs in the testing set. In actual severe cases, ten cases were predicted as mild-moderate. These images were classified into the severe class because the visual field test demonstrated the defective points within the central five degrees. However, the model predicted them into a mild-moderate class due to the cup-to-disc ratio being 0.7–0.8. In actual mild-moderate cases, five were detected as severe because the cupping was large, but the visual field demonstrated moderate severity. Eight cases were detected as normal due to the small cupping; however, mild scotoma in the visual field test was observed. In the latter group, the functional defect of glaucoma occurred prior to the structural defect. In actual normal cases, three were predicted as mild-moderate. We assumed that the model classified the images into this class due to the physiologically large cupping of the disc. Last but not least, one case in the actual severe class was predicted as normal. We tracked this photograph and found that the ONH was pale, and the cup-to-disc ratio was hardly defined due to the saucerized morphology of the disc. Nevertheless, the cupping to the rim, pallor disc, and large PPA correspond with severe glaucoma. The majority of these misclassified images could be explained by the complicated relationship between structure and function in glaucoma.

Certain factors affect the performance of the model. Since HPA classification relies on both mean deviation and pattern deviation (PD), any factors precluding the visual field evaluation could affect the test results. Lens opacity from cataracts might affect the PD value, especially in cases of severe glaucoma. The PD value might overestimate the glaucomatous visual field damage as the cataract progresses^20,21^. This error might not be shown as an unreliable visual field test. Variability of the raw fundus photographs is another factor that might affect model performance. Some photographs were taken from patients with dilated pupils, resulting in variability in the brightness and intensity of the images, although the images were obtained from the same fundus camera.

In our study, we incorporated vertical flipping as part of the data augmentation strategy. While previous studies have primarily utilized horizontal flipping and rotation for augmenting fundus photographs, vertical flipping has been less commonly applied. Notably, a recent study^19^ included vertical flip among its augmentation methods. We believed that glaucomatous changes in optic disc morphology may originate in either the superior or inferior hemispheres, depending on the orientation of the nerve fiber layer along the X-axis. By interchanging the superior and inferior regions of the image, vertical flipping enriches the dataset and introduces additional variability. This augmentation technique has the potential to enhance the detection of glaucomatous optic neuropathy and improve the training efficacy of CNN models by preventing overfitting^22^ and minimizing sensitivity to image alignment^23^. Importantly, we considered this approach to be consistent with the pathophysiology of glaucoma and suitable for clinical application.

We aimed to evaluate the performance of the deep learning model using a single fundus photograph. Since fundus cameras are widely available in most primary care hospitals in Thailand, primarily for diabetic retinopathy screening, this approach holds considerable promise for glaucoma screening, especially in non-ophthalmic settings. Using a single imaging modality reflects real-world clinical practice, providing benefits in the routine practice of glaucoma screening. Although multimodal algorithms have demonstrated high performance, some studies suggested that the model trained using a single fundus photograph is not inferior to those developed using multi-modality inputs^10,19^.

Previous studies have primarily focused on binary classification models for glaucoma detection, demonstrating high performance for ML models^10,12,13,19,24^. Additionally, we developed a model for ternary glaucoma classification. In addition to detecting glaucoma, this model helps us in differentiating mild-moderate and severe stage of the disease. This distinction is clinically important, as severe glaucoma, if left untreated, can lead to rapid and irreversible visual loss. Patients detected as having severe glaucoma require faster confirmation of their diagnosis than those detected as having mild-moderate glaucoma.

There are some limitations to the study. Firstly, fundus photographs of mild and moderate classes were grouped together. Initially, we intended to classify the disease severity into four classes. However, because our hospitals are tertiary care centers that received a high volume of referrals for severe glaucoma, the dataset became imbalanced, with disproportionately fewer images in the mild and moderate classes. Accordingly, we decided to combine the mild and moderate classes together and train the model using three groups. We considered that the inability to classify mild and moderate stages would not affect the purpose of the study, which was to screen for glaucoma and to identify patients at a severe stage who require urgent intervention. Secondly, as previously mentioned, the model was trained using a single imaging modality. To enhance the model’s diagnostic performance, we plan to incorporate multimodal data, including OCT parameters, in the future work. Additionally, to address the issue of false positives arising from discrepancies between cup-to-disc ratio and visual field results, we propose exploring advanced feature encoding strategies within the EfficientNetB7 architecture. We anticipate that these approaches will improve the model’s specificity and overall clinical utility. Lastly, the HPA criteria may overestimate or underestimate glaucomatous damage in some conditions. These gold standard criteria have been used to classify glaucoma severity in our clinical practice and research studies attributable to the clarity and reproducibility of the grading system, and we, therefore, considered it suitable for this study. Also, the criteria are less likely to underestimate the severity of glaucomatous damage than simpler global indices^25^. Other glaucoma classification methods, such as visual field grading using AGIS or CIGTS scores, are recommended for assessing glaucoma severity in future studies aimed at improving model performance.

In conclusion, this study proposes a high-performance machine learning model for classifying normal and glaucoma severity based on the HPA criteria. The model requires only a single fundus photograph, making it particularly suitable for screening in resource-limited settings where access to comprehensive modalities is restricted. This deep learning technology will be helpful to ophthalmologists and other medical personnel without specialized training in glaucoma diagnosis, thereby enabling earlier detection and more effective management of this preventable disease.

## Electronic supplementary material

Below is the link to the electronic supplementary material.

Supplementary Material 1

Supplementary Material 2

## Data Availability

The datasets generated during and/or analyzed during the current study are available in the OSF database repository, https://osf.io/jc2gq/?view_only=0a3ce5873b334064a3af17bbc26ea92e.

## References

[1] Tham, Y. C. et al. Global prevalence of glaucoma and projections of glaucoma burden through 2040: a systematic review and meta-analysis. *Ophthalmology* **121**, 2081–2090 (2014). 24974815 10.1016/j.ophtha.2014.05.013

[2] Li, F. et al. Automatic differentiation of glaucoma visual field from non-glaucoma visual filed using deep convolutional neural network. *BMC Med. Imaging*. **18**, 35 (2018). 30286740 10.1186/s12880-018-0273-5 PMC6172715

[3] Li, Z. et al. Efficacy of a deep learning system for detecting glaucomatous optic neuropathy based on color fundus photographs. *Ophthalmology* **125**, 1199–1206 (2018). 29506863 10.1016/j.ophtha.2018.01.023

[4] Liu, H. et al. Development and validation of a deep learning system to detect glaucomatous optic neuropathy using fundus photographs. *JAMA Ophthalmol.* **137**, 1353–1360 (2019). 31513266 10.1001/jamaophthalmol.2019.3501 PMC6743057

[5] Seo, S. B. & Cho, H. K. Deep learning classification of early normal-tension glaucoma and glaucoma suspects using bruch’s membrane opening-minimum rim width and RNFL. *Sci. Rep.* **10**, 19042 (2020). 33149191 10.1038/s41598-020-76154-7 PMC7643070

[6] Oh, S., Park, Y., Cho, K. J. & Kim, S. J. Explainable machine learning model for glaucoma diagnosis and its interpretation. *Diagnostics* **11**, 510 (2021). 33805685 10.3390/diagnostics11030510 PMC8001225

[7] Shin, Y. et al. Deep learning-based diagnosis of glaucoma using wide-field optical coherence tomography images. *J. Glaucoma*. **30**, 803 (2021). 33979115 10.1097/IJG.0000000000001885

[8] Schottenhamml, J. et al. Glaucoma classification in 3 x 3 mm en face macular scans using deep learning in a different plexus. *Biomed. Opt. Express*. **12**, 7434–7444 (2021). 35003844 10.1364/BOE.439991 PMC8713669

[9] Wu, J. H., Nishida, T., Weinreb, R. N. & Lin, J. W. Performances of machine learning in detecting glaucoma using fundus and retinal optical coherence tomography images: a meta-analysis. *Am. J. Ophthalmol.* **237**, 1–12 (2022). 34942113 10.1016/j.ajo.2021.12.008

[10] Xiong, J. et al. Multimodal machine learning using visual fields and peripapillary circular OCT scans in detection of glaucomatous optic neuropathy. *Ophthalmology* **129**, 171–180 (2022). 34339778 10.1016/j.ophtha.2021.07.032

[11] Cho, H. et al. Deep learning ensemble method for classifying glaucoma stages using fundus photographs and convolutional neural networks. *Curr. Eye Res.* **46**, 1516–1524 (2021). 33820457 10.1080/02713683.2021.1900268

[12] Noury, E. et al. Deep learning for glaucoma detection and identification of novel diagnostic areas in diverse real-world datasets. *Transl Vis. Sci. Technol.* **11**, 11 (2022). 35551345 10.1167/tvst.11.5.11 PMC9145034

[13] Hung, K. H. et al. Application of a deep learning system in glaucoma screening and further classification with colour fundus photographs: a case control study. *BMC Ophthalmol.* **22**, 483 (2022). 36510171 10.1186/s12886-022-02730-2 PMC9743575

[14] Valente, C., D’Alessandro, E. & Iester, M. Classification and statistical trend analysis in detecting glaucomatous visual field progression. *J. Ophthalmol.* **2019**,1583260 (2019). 10.1155/2019/1583260 PMC6558616 31275629

[15] Chakravarti, T. Assessing precision of Hodapp-Parrish-Anderson criteria for staging early glaucomatous damage in an ocular hypertension cohort: a retrospective study. *Asia-Pac J. Ophthalmol.* **6**, 21 (2017). 10.1097/APO.0000000000000201 28161915

[16] Anderson, D. R. & Patella, V. M. *Automated Static Perimetry* (Mosby Inc, 1999).

[17] Tan, M. & Le, Q. V. EfficientNet rethinking model scaling for convolutional neural networks. *Preprint At.*10.48550/arXiv.1905.11946 (2020).

[18] Deng, J. et al. ImageNet: a large-scale hierarchical image database. In *2009 IEEE Conference on Computer Vision and Pattern Recognition* 248–255 (2009).

[19] Xue, Y. et al. A multi-feature deep learning system to enhance glaucoma severity diagnosis with high accuracy and fast speed. *J. Biomed. Inf.* **136**, 104233 (2022). 10.1016/j.jbi.2022.104233 36280089

[20] Matsuda, A. et al. Do pattern deviation values accurately estimate glaucomatous visual field damage in eyes with glaucoma and cataract? *Br. J. Ophthalmol.* **99**, 1240–1244 (2015). 25795915 10.1136/bjophthalmol-2014-306019

[21] Tomita, R. et al. Accuracy of pattern deviation in estimating the glaucomatous damage in the central 10° visual field in eyes with glaucoma and cataract. *Br. J. Ophthalmol.* **108**, 78–83 (2023). 36261260 10.1136/bjo-2022-322274

[22] Kim, S., Yun, W., Oh, J. & Lee, S. Efficient deep learning approaches for processing ultra-widefield retinal imaging. In *MICCAI Challenge on ultra-widefield fundus imaging for diabetic retinopathy, Marrakesh, Morocco, October 10, 2024. Proceedings* (ed. Sheng, B.) 125–134 (Springer, 2025).

[23] Akhtar, S. et al. A deep learning based model for diabetic retinopathy grading. *Sci. Rep.* **15**, 3763 (2025). 39885230 10.1038/s41598-025-87171-9 PMC11782675

[24] Sudhan, M. B. et al. Segmentation and classification of glaucoma using U-Net with deep learning model. *J. Healthc Eng.* **2022**, 1601354 (2022). 10.1155/2022/1601354 PMC8866016 35222876

[25] Thirunavukarasu, A. J. et al. A validated web-application (GFDC) for automatic classification of glaucomatous visual field defects using Hodapp-Parrish-Anderson criteria. *Npj Digit. Med.* **7**, 1–4 (2024). 38762669 10.1038/s41746-024-01122-8 PMC11102533

