# Supplementary material for: Machine learning technology in the classification of glaucoma severity using fundus photographs
Source: Sci Rep. 2025 Jul 18;15:26151. doi: 10.1038/s41598-025-11697-1 (PMC12274414; doi:10.1038/s41598-025-11697-1)
Supplement: Supplementary file 1 — Supplementary Material 1 [file 41598_2025_11697_MOESM1_ESM.pdf]

Supplementary Data S1 Hodapp-Parrish-Anderson Classification

Mild defect: meet all requirements

1. The MD is better than -6 dB;
2. Fewer than 18 or 76 points in a 30-2 pattern (25%) are defective in the total deviation probability plot at the 5% level;
3. Fewer than 10 points are defective at the 1% level; AND
4. No point in the central 5 degrees has a sensitivity less than 15 dB.

Moderate defect: exceed one or more of the criteria required to keep it in the early defect category but does not meet the criterion to be severe.

Severe defect: any of the following

1. An MD index worse than -12 dB;
2. More than 37 (50%) of the points depressed at the 5% level;
3. More than 20 points depressed at the 1% level;
4. A point in the central 5 degrees with 0-dB sensitivity; OR
5. Points closer than 5 degrees of the fixation under 15-dB sensitivity in both the upper and lower hemifields.
