# Supplementary material for: Machine learning technology in the classification of glaucoma severity using fundus photographs
Source: Sci Rep. 2025 Jul 18;15:26151. doi: 10.1038/s41598-025-11697-1 (PMC12274414; doi:10.1038/s41598-025-11697-1)
Supplement: Supplementary file 2 — Supplementary Material 2 [file 41598_2025_11697_MOESM2_ESM.pdf]

Supplementary Fig. S2 Characteristics of Misclassified Fundus Photographs

Actual	Predict	Number	Example photographs	Explanation
Severe	Mild-moderate	10	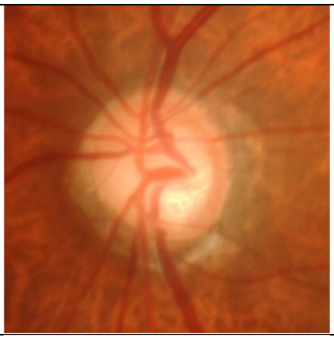	There are defective points within the central 5 degrees of the automated visual field test.
Mild-moderate	Severe	5	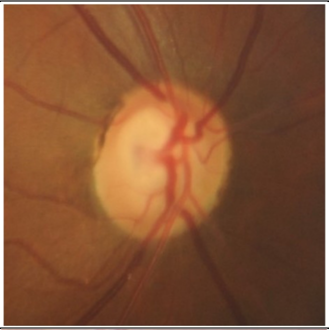	Structural loss detected by fundus photograph happened prior to functional loss detected by visual field test.
Mild-moderate	Normal	8	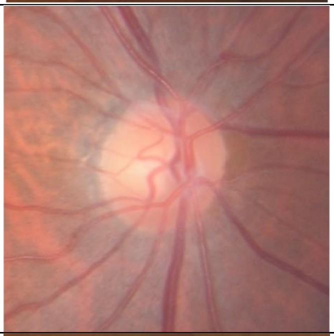	Early glaucoma presents with small cupping and mild visual field defect.
Normal	Mild-moderate	3	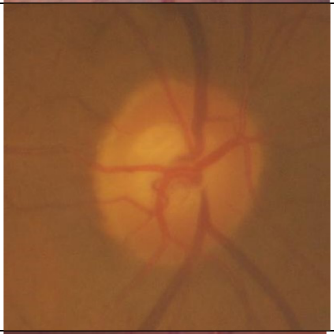	Physiologic large cupping
Severe	Normal	1	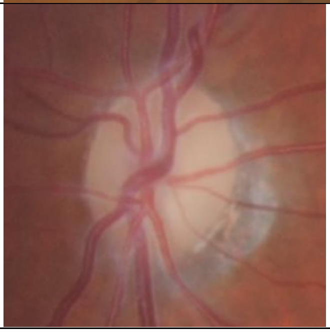	Shallow cupping with pale disc
